# Research protocol for impact assessment of a project to scale up food policies in the Pacific

**DOI:** 10.1186/s12961-022-00927-x

**Published:** 2022-10-29

**Authors:** Dori Patay, Kathy Trieu, Briar McKenzie, Shanthi Ramanathan, Alexis Hure, Colin Bell, Anne-Marie Thow, Steven Allender, Erica Reeve, Aliyah Palu, Mark Woodward, Gade Waqa, Jacqui Webster

**Affiliations:** 1 The George Institute for Global Health, Level 5, 1 King Street, Newtown, Sydney, NSW 2042 Australia; 2 Hunter Medical Research Institute, School of Medicine and Public Health, University of Newcastle, Lot 1 Kookaburra Cct, New Lambton Heights, NSW 2305 Australia; 3 Global Centre for Preventive Health and Nutrition (GLOBE), Institute for Health Transformation, Deakin University, Geelong, VIC Australia; 4 Charles Perkins Centre, Faculty of Medicine and Health, University of Sydney, John Hopkins Dr, Camperdown, NSW 2006 Australia; 5 The George Institute for Global Health, School of Public Health, Imperial College London, 84 Wood Lane, London, W12 0BZ UK; 6 Pacific Research Centre for the Prevention of Obesity and Non Communicable Diseases, Fiji National University, Princess Road, Tamavua, Suva, Fiji

**Keywords:** Research impact assessment, Research translation, Process evaluation, Food policy, Fiji, Pacific health, Implementation science

## Abstract

**Background:**

One of the challenges for countries implementing food policy measures has been the difficulty in demonstrating impact and retaining stakeholder support. Consequently, research funded to help countries overcome these challenges should assess impact and translation into practice, particularly in low-resource settings. However, there are still few attempts to prospectively, and comprehensively, assess research impact. This protocol describes a study co-created with project implementers, collaborative investigators and key stakeholders to optimize and monitor the impact of a research project on scaling up food policies in Fiji.

**Methods:**

To develop this protocol, our team of researchers prospectively applied the Framework to Assess the Impact from Translational health research (FAIT). Activities included (i) developing a logic model to map the pathway to impact and establish domains of benefit; (ii) identifying process and impact indicators for each of these domains; (iii) identifying relevant data for impact indicators and a cost–consequence analysis; and (iv) establishing a process for collecting quantitative and qualitative data to measure progress. Impact assessment data will be collected between September 2022 and December 2024, through reports, routine monitoring activities, group discussions and semi-structured interviews with key implementers and stakeholders. The prospective application of the protocol, and interim and final research impact assessments of each project stream and the project as a whole, will optimize and enable robust measurement of research impact.

**Discussion:**

By applying this protocol, we aim to increase understanding of pathways to impact and processes that need to be put in place to achieve this. This impact evaluation will inform future projects with a similar scope and will identify transferable and/or translatable lessons for other Pacific Island states and low- and middle-income countries.

**Supplementary Information:**

The online version contains supplementary material available at 10.1186/s12961-022-00927-x.

## Background

Demonstrating impact and retaining stakeholder support is essential for food policy implementation [1]. Poor diet is a major driver of noncommunicable disease (NCD) [2], and evidence of the benefits of food policy interventions on improving population diet has been growing [3–6]. Eighty percent of NCD deaths globally occur in low- and middle-income countries (LMICs) [7], crippling countries’ efforts to reach their Sustainable Development Goals (SDGs). Insufficient resources also thwart these efforts, requiring researchers to test implementation strategies that fight NCDs as the primary cause of mortality and morbidity and maintain evidence of the impact of these strategies.

To this end, researchers should demonstrate the impact of their research and report translation into practice. Research impact assessments are often used to retrospectively measure health research outcomes, after the period where nuanced and reliable information can be gathered [8, 9], and process evaluations are often incorporated into projects to help understand mechanisms of change and factors that influence implementation [10]. While the retrospective application of these methods can demonstrate research impact, prospective application can also optimize research impact by enabling the systematic planning, monitoring and continuous improvement of project implementation [9, 10]. Yet, few research projects comprehensively plan to optimize and monitor their impact [8, 11–14].

NCDs have slowed economic development in Pacific Island countries and territories (PICs), holding them back from reaching their Healthy Islands vision, according to health ministers [15], and from achieving SDGs [16, 17] by at least a decade. PICs are committed to improving population diet [15], but face challenges implementing policy [18]. Barriers to implementation include capacity constraints, logistical and operational challenges, low political support, limited multisectoral collaboration and lack of context-specific evidence [19–24].

Translational research projects are needed to test strategies for implementing and scaling up food policies. Further, the effectiveness of such projects needs to be measured [10]. However, the literature on research impact assessments and process evaluations primarily focuses on high-income countries [8, 11–13, 25–27]. Given the increasing burden of NCDs and the struggle to implement effective nutrition policies in PICs, there is an urgent need to expand evidence on the ways translational research projects can benefit food policy implementation.

This paper introduces a collaboratively developed protocol to optimize the impact of an implementation research project, “Scaling-Up food Policy Interventions to reduce noncommunicable diseases in the Pacific Islands” (henceforth SUPI). SUPI—funded by the Global Alliance for Chronic Diseases—aims to strengthen and monitor food policy interventions in Fiji and was designed as a pragmatic type 3 implementation effectiveness hybrid trial [28]. SUPI consists of four project streams: a policy landscape analysis to map existing policy content, stakeholders, and politics (Stream 1); an economic analysis to support policy development and adoption with the focus on the impact and cost of salt reduction strategies and sugar-sweetened beverage taxes (Stream 2); a collaborative process to strengthen policy development and implementation to engage stakeholders to identify, implement and monitor actions to strengthen food policy interventions (Stream 3); and repeated cross-sectional surveys before and after the supported interventions to assess their impacts through routine monitoring of dietary intake, diet behaviours and nutrition composition of processed packaged food supply (Stream 4).

## Methods

This protocol paper describes our approach to optimize and monitor the research impact of SUPI on scaling up food policies in Fiji through the prospective application of a research impact assessment. SUPI originally included both Fiji and Samoa; however, due to COVID-19-related changes in priorities in Samoa, it is now primarily focused on Fiji. The research team consists of researchers from Fiji National University, The George Institute for Global Health, Deakin University and the University of Sydney. Participants in this research impact assessment will be key project implementers (researchers and research assistants) and SUPI Reference Group members, such as representatives of the Ministry of Health (and other relevant government agencies) of Fiji, the Secretariat of the South Pacific, WHO, the World Bank, the United Nations Food and Agricultural Organization and other key stakeholders already involved in the project, such as the Fiji Consumer Council.

The evaluation of each SUPI research project stream will be conducted separately, supplemented by an overall programme evaluation. Table 1 presents the timeline for the implementation and evaluation of each project stream and the project overall. In addition to the final research impact assessment of each project stream and the overall SUPI, an interim research impact assessment will be conducted for Stream 3, the selected interventions and for the overall SUPI. The prospective application of this protocol and the interim assessments support comprehensive and careful planning, implementation and monitoring of research activities of SUPI and, as such, are designed to help the project achieve optimal research impact.

**Table 1:** The timeline of the research impact assessment

SUPI Stream name	Timeline			
	Implementation	Research impact assessment		
		Data collection	Analysis	Dissemination of findings
Stream 1 Policy landscape analysis	March 2020–June 2021	September 2022–February 2023	February–March 2023	April–June 2023
Stream 2 Economic analysis	Impact and cost of salt reduction: July–December 2020. Impact and cost of sugar-sweetened beverage taxes: August 2021–May 2022	September 2022–February 2023	February–March 2023	April–June 2023
Stream 3 Collaborative process with policy-makers	September 2022–September 2024	Interim assessment: March–June 2023 Final assessment: September–October 2024	July–September 2023	October–December 2023
Stream 4 Nutrition surveys	Nutrition composition of food products: baseline assessment in September 2020–July 2021; second assessment in 2023. Baseline nutrition survey: March–July 2022	September 2022–February 2023	February–March 2023	April–June 2023
SUPI overall	August 2019–December 2024	Interim assessment: March–June 2023 Final assessment: September–October 2024	July–September 2023	October–December 2023

A collaborative approach in the development and application of research impact and process evaluations has been cited as a highly effective way to enhance validity and precision [8–10]. Moreover, it strengthens stakeholder engagement, potentially helping retain stakeholder support. Therefore, during the development of the study protocol, we consulted with key project implementers, Reference Group members and relevant stakeholders (representing the organizations listed above). The interview guide used during the initial consultations is presented in Additional file 1. After the initial discussions, several follow-up meetings were held, where together we worked to adapt the original logic model of SUPI (designed when the project was planned) [28] to help identify intended domains of benefits and process and impact indicators; identify data that would need to be collected to measure impact and for the cost–consequence analysis; and establish a pragmatic mixed-methods process for collecting quantitative and qualitative data.

### The frameworks informing the protocol

We used the Framework to Assess the Impact from Translational health research (FAIT) [8, 9], the Medical Research Council guidelines [10] and earlier process evaluations in food and nutrition policy research [27, 29–31] to design our protocol.

The purpose of prospectively applying research impact assessment methods is to (i) identify pathways to impact from need through to pathways to adoption, (ii) identify intended and aspirational research outcomes and benefits, and (iii) help to plan for and adequately resource translational activity to achieve the intended impacts [8]. FAIT, as developed by the Hunter Medical Research Institute, combines three commonly used methods for impact assessment using a mixed-methods approach: a modification of the original payback model [32], describing and measuring impact using quantitative indicators within the identified domains of impact, depending on the research project; an economic analysis to measure the social return of investment; and a narrative description of research translation and impact [9].

We used FAIT to inform the design of the research impact assessment in our protocol for multiple reasons. First, SUPI requires an approach that can measure change resulting from a wide range of activities, such as policy landscape analysis, economic modelling of interventions, nutrition surveys and collaborative approaches to strengthening policy. The complex nature of this project makes it suited to a combination of quantitative and qualitative methods [8, 9]. Second, FAIT was specifically developed to inform translational health research [8, 9], which aligns with SUPI, since its aim is to translate nutrition research into policy and practice. Third, the application of FAIT for nutrition research in PICs has already been trialled in a prior study [8], with direct relevance to SUPI.

To ensure ongoing monitoring to assess implementation and ensure that the impact goals of SUPI are being reached, a process evaluation has been integrated into our protocol. A process evaluation aims to expand the understanding of how and why the outcomes of a research project were achieved, how and why the project worked or did not work, and document experiences and lessons for translation [10]. The Medical Research Council guidance [10] and earlier process evaluations conducted in the Asia Pacific region in food and nutrition policy research [27, 29–31] have informed our process evaluation design. We drew from Linnan and Steckler’s framework [33] and the RE-AIM (Reach, Effectiveness, Adoption, Implementation, Maintenance) framework [34] to assess policy implementation through seven analytical constructs: fidelity, dose, reach, effectiveness, adoption, context and cost. Table 2 presents the definition of each construct. We chose this approach for process evaluation because it has been successfully utilized to assess a complex nutrition intervention in a PIC [30]. Recognizing that *context* is a large domain, potentially encompassing a range of interest-based, ideational and institutional factors, the data analysis will allow for inductive interpretation of the emerging themes within this domain.

**Table 2:** The analytical constructs of the process evaluation

Analytical constructs	Definition
Fidelity [33]	Degree to which the research project components were delivered as planned [10, 33]
Dose [33]	Extent participants actively engaged with the research project component [10, 33]
Reach [33]	Number or proportion of the intended target audience that comes into contact with the research project component [33, 34]
Effectiveness [34]	Positive and negative impacts of the research project component [34]
Adoption [34]	Proportion/representativeness of organizations adopting the intervention [27, 34]
Context [33]	Political, sociocultural, economic, commercial and other factors impacting the implementation of the research project components [10]
Cost (30)	The cost of the research project component

### Identifying domains of benefits, process and impact indicators

**Fig. 1:**
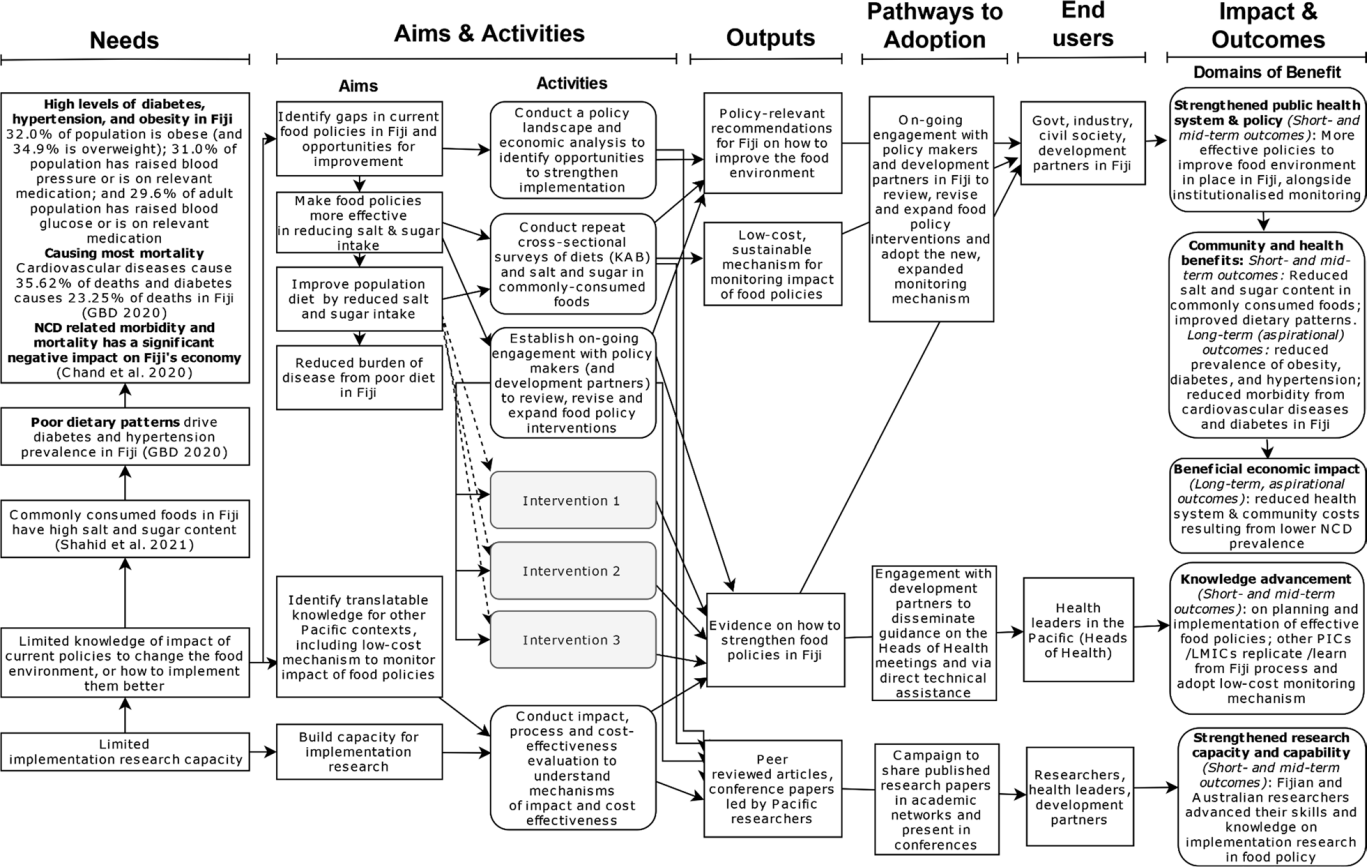
The logic model of the SUPI research project based on the integrated framework to optimize research impact

The logic model of SUPI [28]—identifying the research outputs, outcomes and pathways to adoption—has been updated to capture the impact of contextual factors on project implementation and outcomes to date (Fig. 1). Table 3 shows a detailed list of potential benefits and corresponding metrics to measure each outcome identified in the logic model (last column). A detailed list of input, process, output and outcome indicators informing the research impact and process evaluation according to each SUPI project stream is provided in Additional file 2.

**Table 3:** The domains of benefits, metrics and indicators

Domain of benefit (identified from the logic model)	Metric	Indicator
Community and health benefits	Consumer knowledge and awareness of health risks associated with salt and sugar consumption	% of improvement in knowledge
		% of improvement in attitudes
		% of improvement in behaviour
	Reduced affordability of processed foods (*depending on intervention*)	% of absolute/relative change in processed food prices
	Reduced availability of processed foods (*depending on intervention*)	# of high-sodium/high-sugar products readily available in schools, supermarkets, workplaces
	Reduced consumption of processed foods (*depending on intervention*)	% reduction in processed foods consumption
	Product reformulation (*depending on intervention*)	% decrease in sodium content of processed food products
		% decrease in sugar content of processed food products
	Reduction in salt/sugar intake	% reduction in daily sodium intake
		% reduction in daily sugar intake
Public health system and policy strengthening	Adoption of a new system for monitoring food policy impact in Fiji	New components adopted in the monitoring activity included in Ministry of Health and Medical Services plans
	Adoption of new targets based on new data sets in Fiji	Inclusion of sodium and sugar intake target levels with deadlines in Ministry of Health and Medical Services plans
	Changes to current food policies in Fiji	# of changed policies or plans (aspirational)
	New school policies on food implemented in Fijian schools (*depending on intervention*)	# of schools implementing new (or existing) food policies to target the consumption and availability of unhealthy foods in schools
Economic impact	Current and future income of staff associated with the study	Amount of research team wages contributed to the Fijian economy: (total wages)
		# of Fijian research staff receiving wage for participation in this project
		Amount of research team wages contributed to the Australian economy: (total wages)
		# of Australian research staff receiving wage for participation in this project
		# of jobs created in Fiji
		# of jobs maintained in Fiji
		# of jobs created in Australia
		# of jobs maintained in Australia
		Amount of additional lifetime income of Ph.D. students
	Reduced health system costs and generated revenues (*depending on intervention*)	Amount of revenue generated with x% increase in SSB tax (economic modelling of SSB taxes in Fiji) (could be economic or policy/legislation)
		Amount saved with implementing x interventions (economic modelling of salt reduction strategies in Fiji)
	Reduced spending on processed foods	Estimate can be calculated based on price data and % of consumption change, estimated for the population based on the cohort data
	New research financing	Amount of new research funds gained (leveraged funding)
Knowledge advancement	New data (sets)	# of new data sets
		# of users who use the new data
		# of times new data was used as evidence in writing
	Publications (publicly available)	# of peer-reviewed research articles
		# of other publications (publicly available)
		# of citations
		# of downloads
		# of reads
		# of Altmetric score
	Newsletters	# of newsletters
		# of individuals received the newsletters
	Results briefs and technical documents	# of results brief and technical documents
		# of individuals received the briefs and technical documents
		# of users of technical documents
	Presentations, webinars, workshops	# of presentations in international/regional conferences
		# of presentations in national conferences
		# of workshops for international/regional audience
		# of workshops for national audience
		# of attended audience
	Media and social media	# of media mentions
		# of mentions on Twitter
		# of mentions on Facebook
		# of Twitter likes and comments
		# of Facebook likes or comments
		# of website views
Research capacity and capability-building	Academic qualifications	# of Fijian students associated with the project
		# of Australian students associated with the project
		# of master’s degrees earned by Fijian/Samoan staff members
		# of Ph.D. degrees earned by Fijian staff members
	Researchers and research assistants	# of researchers and research assistants with capacity built in implementation science (any aspect of the project)
	Knowledge and capabilities in developing and implementing food and nutrition policies	# of Fijian researchers and government officials participated in any aspect of the project
	New research network	# of Fijian staff who are co-authors on peer-reviewed papers
		# of Fijian staff who are collaborators on future grants
		# of co-applications for future grants

The updated logic model of SUPI guided the identification of the domains of benefits, in which the research impact will be assessed: knowledge advancement, research capacity and capability-building, public health system and policy strengthening, community and health benefits, and economic impact (Table 3). Each domain contains multiple items with metrics allowing quantitative measurements of change. For example, within the knowledge advancement domain, “new data sets” is listed as one of the metrics, accompanied by measurable indicators such as the number of new data sets or the number of times new data were used as evidence in writing. In addition, input and process indicators were identified for each of the four streams to monitor and provide feedback on the implementation process. Costing data will involve a log of all intervention activities including the individual’s involved, their roles and wages and the time taken for implementation. Other resources such as travel and consumables will also be costed. Qualitative data will be collected through interviews and group discussions where questions about each domain of impact, the mechanisms of change and the implementation of the project streams will be asked.

### Identifying cost data for the economic analysis

To measure whether the cost associated with SUPI and the use of its outcomes are worth the benefits and consequences achieved, a cost–consequence analysis will be undertaken [35–37]. This economic evaluation method is recommended for complex projects with multiple effects that are hard to monetize and reduce to a single measurement outcome such as a cost–benefit ratio [35–37]. Furthermore, given restrictions in funding and limited availability of health economists, cost–consequence analysis is less resource intensive and useful when a full cost–benefit analysis is premature [35]. It also allows for outcomes to be valued in their natural units which is already covered by the payback analysis, further streamlining the assessment process.

First, we will collect the cost of implementing each of the four project streams. This can be prospectively calculated based on the budget plan. Second, the cost of implementing the proposed interventions will be calculated. In the case of SUPI, the interventions will be designed based on the policy landscape analysis (Stream 1), economic modelling (Stream 2) and nutrition surveys (Stream 4). Government officials will decide on which intervention to pursue during the collaborative process to strengthen policy development and implementation (Stream 3); therefore, these calculations will be conducted after implementing these research project streams. In addition, the cost of maintaining the delivery of the chosen interventions (including the cost to society, government and industry) will be collected and added to the overall cost, which will be calculated concurrently to the intervention costs.

### Data collection

Data collection to evaluate each project stream will be conducted separately within the timeline presented in Table 1. Data collection to assess the selected interventions will be conducted in 2023 and 2024, once they have been implemented. Data for the research impact assessment will be collected in an integrated manner through the application of the following four methods.

#### Routine monitoring of implementation embedded into each project stream

The purpose of this data collection method is to collect quantitative data to monitor and measure the research impact of SUPI and its implementation progress. The data will be collected online or via email by accessing the project records. Data will be collected from the routine monitoring and implementation tracking records of each project stream. For example, these records include the publication and conference presentation tracking Excel file used to monitor all dissemination activities related to the project, budget projection and actual spending records, or implementation records of number of participants and stakeholders involved in the different project streams.

#### Reports during the regular team meetings

This data collection method aims to collect quantitative data to monitor and measure the implementation progress and impact of SUPI that are not recorded during routine monitoring. The data will be collected online by accessing the recorded meeting minutes.

#### Semi-structured interviews with SUPI staff, collaborative investigators, Reference Group members and other key stakeholders

Our aim with this data collection method is to collect qualitative data to understand the research impact of SUPI, how and why was this research impact achieved, and what are the lessons for continuation and/or replication of the food and nutrition policies in other PICs or LMICs. Moreover, the aim of these interviews is to understand the extent to which each project stream was implemented, the barriers and facilitators of implementation, and lessons for the future to help the implementation of similar projects. Thus, the interviews collect data for the process evaluation component, besides the focus on research impact. The interviews will be conducted face to face or online, and they are expected to take 30–90 minutes. This time range reflects participants have more or less to contribute, and interviews in earlier process evaluations in PICs showed similar duration [29, 30]. The interview questions will be open-ended and semi-structured, informed by the research impact domains identified in the logic model (Fig. 1) and the constructs listed in the analytical framework for the process evaluation (Table 2). The interview guide is provided in Additional file 3. Where interviewees prefer anonymity, their identity can be kept confidential by means of a cover ID.

#### Group discussion during the biannual leadership team meetings

The purpose of this data collection method is to collect qualitative data to understand the research impact of SUPI, how and why was this research impact achieved, and what are the lessons for continuation and/or replication of the food and nutrition policies in other PICs. The group discussions focus on planning and understanding the research impact of SUPI, but do not investigate issues within the scope of the integrated process evaluation. The group discussions will be conducted face to face or online, and they are expected to take 90–120 minutes, depending on the involvement of the participants [29, 30]. The questions asked will be open-ended and semi-structured, informed by the research impact domains identified in the logic model (see Fig. 1). The guide for the group discussions is provided in Additional file 4.

To minimize the burden and streamline the data collection process, a data collection card was developed in a Microsoft Excel file for each project stream and for the overall SUPI that includes all input, process, output and outcome indicators relevant to the given stream components. The data collection cards for the project streams and for the overall SUPI are presented in Additional file 2. Automated links connect the data in the data collection cards to a research impact assessment quantitative summary score card (containing the metrics presented in Table 3) and a separate summary process evaluation score card; thus, data need to be entered only once. This pragmatic data collection approach enables the collection of a large amount of quantitative data while minimizing administrative burden and simplifying data analysis. The research impact assessment quantitative summary score card is presented in Additional file 5.

### Data analysis

#### Quantitative analysis

Where applicable and appropriate, descriptive statistics (mean and standard deviation for continuous variables and frequency and proportion for categorical variables) will be used to summarize quantitative data (see Table 3). The economic analysis will involve monetization of the research costs and implementation costs of the selected interventions using standard economic techniques including the addition of oncosts and overheads to all labour costs and converting and presenting costs in 2024 Australian dollars. Where appropriate and possible, a monetization of the benefits and consequences will involve application of published costs, such as the cost of hospitalizations from acute cardiovascular incidents. To assist with understanding the latent benefits, projections underpinned by clear and transparent assumptions will be used to model the future impacts of the interventions, and sensitivity analysis and attribution will be used to derive conservative estimates of the potential value of future benefits. All non-monetizable consequences will be listed in their natural units and displayed within the payback results. Unlike a cost–benefit analysis, no attempt will be made to present a single ratio of the investment versus the returns. Valuation of the social return on investment will rest with the reader who can make their own judgement based on both the monetizable and non-monetizable benefits.

#### Qualitative analysis

The qualitative data will be transcribed by an independent company, and de-identified transcripts will be uploaded to NVivo software, where it will undergo deductive and inductive coding. The primary nodes used for the coding will be the domains identified in the logic model (see Fig. 1) and in the analytical constructs of the process evaluation (see Table 2). This will be supplemented by inductively identified subcodes as relevant, to allow the emergence of new patterns or important themes from the data.

The data will be triangulated in three ways: (i) methods triangulation, through the combination of quantitative, qualitative and economic methods; (ii) triangulation of sources, by interviewing participants with different roles and overview of each research project stream; and (iii) analyst triangulation, with several researchers reviewing and interpreting the results [38].

### Dissemination

The progress, interim findings and results of the process evaluation will be reported to the project staff and Reference Group members. The interim results will allow adjustments in implementing the research project components, ensuring the most optimal outcome, and they will help retaining stakeholder support through regular engagement. In addition, academic papers will be written and submitted for peer review, presentations will be held in Pacific-focused and international conferences, and a Fiji National University newsletter will be produced to share the final results. Finally, the findings will be shared with PIC governments via regional intergovernmental events, such as Heads of Health meetings, and with Secretariat of the South Pacific, WHO, the Food and Agricultural Organization of the United Nations and the World Bank.

## Discussion

### Key contributions

This protocol paper provides an example of how the research impact of a complex, collaborative research project to scale up food policy interventions in PICs can be optimized and monitored, through the prospective application of a pragmatic, mixed-methods approach. The interim research impact (and process evaluation) data will be used to strengthen the implementation of SUPI and help maintain stakeholder support, and the final evaluation will contribute to understanding the enablers and barriers of implementing research projects aiming to scale up nutrition policies in PICs; the benefits such projects can bring and the pathways to impact. This evaluation will inform future projects with similar scope and will identify transferable and/or translatable lessons for other PICs and LMICs. Thus, this study protocol provides important contribution to the public health and translational research scholarship, and support policy-makers in PICs in their efforts to implement nutrition policies.

### Strengths and limitations

The research impact protocol introduced in this paper has several strengths. First, its prospective application supports the proactive planning and implementation of SUPI for optimal research impact, through the development of the logic model that helps in identifying the pathways of impact and the domains of benefits. This proactive approach allows the responsive adaptation of the project implementation to external conditions, such as the COVID-19 pandemic [28]. For example, COVID-19-related changes in priorities in Samoa have resulted in SUPI focusing primarily on Fiji, and the pace of the project implementation has been adjusted to the current capacities in Fiji [28]. Second, the collaborative protocol development process supports stakeholder engagement from the early stages of the study, potentially helping to retain stakeholder support throughout SUPI. Third, feeding into the first strength, the process evaluation embedded into the research impact assessment enables the measurement and evaluation of the progress of SUPI during implementation. This enables course correction to ensure the project can achieve the projected impacts. The indicators—that translate the domains of impact—enable measuring the change resulting from each project stream and the overall project. Moreover, the prospective establishment of such indicators supports the use of data collected through routine monitoring. Fourth, this protocol offers a pragmatic approach to collect and record research impact and process evaluation data within one database, thus simplifying data collection and analysis. Fifth, this research impact assessment helps to implement a multidisciplinary approach to strengthening food policy by ensuring that epidemiology, health education, environmental health, trade and fiscal policy, and in general, food regulation and governance approaches are incorporated within SUPI. Previous research has shown that breaking down sectoral and disciplinary silos is critical for effectively strengthening food policy in PICs [1, 18–23, 39, 40] and LMICs in general [41, 42].

It may, however, be hard to attribute research impact to this particular project. Several projects and programmes are underway in Fiji to strengthen food policy and will influence the outcomes of this research project [43, 44]. Due to this real-world complexity, it will be challenging to accurately determine the contribution of SUPI to each impact, such as change in policy and legislation, or improvement in health outcomes. Attribution and sensitivity analysis will help mitigate this limitation. Moreover, food policy interventions often require a long time to achieve impact; therefore, some of the outcomes are likely to manifest after the completion of SUPI and thus will not be measured by this evaluation protocol. These outcomes are flagged as aspirational in the logic model. As the evaluation protocol will be applied by members of the SUPI research team, there is a risk of bias in data analysis. To mitigate this risk, the evaluation lead will engage with the research project components closely enough to allow a good understanding of the project but remain sufficiently independent to ensure credibility, and independent validation will be provided by two evaluators who are not members of the research team (SR and AH). Finally, the interventions supported by SUPI are context-specific to Fiji; thus, the results on their effectiveness might not be generalizable to other PICs or LMICs [28].

## Conclusions

This protocol paper has demonstrated how the research impact of a complex, multidisciplinary research project to scale up food policies in Fiji can be prospectively planned, optimized and monitored, and the ways stakeholder support can be facilitated. Increasing and measuring the impact of research projects that aim to support PICs’ efforts to scale up food policies is vital to address the NCD epidemic. Translational research projects need to adapt to the changes of contextual factors, such as the COVID-19 pandemic or the global food and energy crisis, and the prospective application of a research impact assessment protocol that enables such responsiveness has the potential to ensure the best possible research outcomes.

## Supplementary Information

**Additional file 1. **Interview guide for the evaluation planning consultations.

**Additional file 2.** Data collection cards for each project stream and SUPI in general.

**Additional file 3.** Interview guide for the research impact assessment.

**Additional file 4.** Topic guide for the group discussions for the research impact assessment.

**Additional file 5.** Research impact assessment quantitative summary score card.

## Data Availability

Not applicable.
