# Supplementary material for: Research protocol for impact assessment of a project to scale up food policies in the Pacific
Source: Health Res Policy Syst. 2022 Oct 29;20:117. doi: 10.1186/s12961-022-00927-x (PMC9617745; doi:10.1186/s12961-022-00927-x)
Supplement: Supplementary file 1 — Additional file 1. Interview guide for the evaluation planning consultations. [file 12961_2022_927_MOESM1_ESM.docx]

Additional File 1.

**Table S1. Interview guide for the evaluation planning consultations**

| **No** | **Question** | **Focus** |
| --- | --- | --- |
| **1** | **Before starting this project stream, what things were* needed to be put in place?** For example: | Input indicators |
|  | funding (financial resources) |  |
|  | staff (human resources) |  |
|  | training (capacity building) |  |
|  | assets (instrumental capacity), e.g. hardware, software, tools, access to databases |  |
|  | collaboration agreed with other project teams |  |
| **2** | **What work activities were needed to be performed to implement this project stream?** For example: | Process indicators |
|  | planning/coordination |  |
|  | data collection: e.g. interviews, databases, surveys, lit reviews, policy reviews |  |
|  | data analysis |  |
|  | reporting |  |
|  | community engagement |  |
|  | stakeholder engagement |  |
| **3** | **What outputs are to be produced in this project stream?** For example: | Output indicators |
|  | new data sets |  |
|  | strengthened local capacity |  |
| **4** | **What activities are needed to be performed after the planned project stream were implemented?** For example: |  |
|  | dissemination |  |
|  | community engagement |  |
|  | stakeholder engagement |  |
| **5** | **What do you plan to achieve directly with this project stream?** | Outcome indicators |
| **6** | **How does this project stream contribute to achieving the project aims?** |  |
| **The tense of interview questions depends on whether the interviews are conducted during or after completing the project tasks.* | | |
