# Supplementary material for: Research protocol for impact assessment of a project to scale up food policies in the Pacific
Source: Health Res Policy Syst. 2022 Oct 29;20:117. doi: 10.1186/s12961-022-00927-x (PMC9617745; doi:10.1186/s12961-022-00927-x)
Supplement: Supplementary file 2 — Additional file 2. Data collection cards for each project stream and SUPI in general. [file 12961_2022_927_MOESM2_ESM.docx]

**Table S2A The policy landscape analysis project stream data collection card (Cs – Cost; D – Dose; R – Reach; F – Fidelity; A - Adoption)**

| **Input indicators** | | **Process indicators** | | | **Output indicators** | | **Outcome indicators** | |  |
| --- | --- | --- | --- | --- | --- | --- | --- | --- | --- |
| **Project Stream Component** | **Input indicator** | **Project Stream Component** | **Process indicator** | **Project Stream Component** | | **Output indicators** | **Project Stream Component** | **Outcome indicators** | |
| Funding | $ of spent budget (Cs) | Planning / Coordination | # of internal reports and briefs (D) | New data and evidence | | Collection of food policy documents (F) | Contributing evidence to policy development, scale-up or strengthening | # of times evidence provided in writing (D) | |
|  | $ spent on wages of Fijian research staff (Cs) |  | # of stakeholders receiving interal briefs ( R) |  |  | Report and summary brief on policy landscape analysis & recommendations, input for collaborative process to strengthen policy development and implementation (internal documents) (F) |  | # policy forums held (D) | |
|  | $ spent on wages of Australian research staff (Cs) |  | development of reporting & stakeholder engagement protocol (F) | Dissemination | | Summary brief of key findings for interview participants/stakeholders (F) |  | # of policy makers involved (R ) | |
| Human resource | # of Fijian staff ( D) |  | development of interview guide (F) |  |  | # of participants/stakeholders receiving the summary brief ( R) |  | # changed policies (aspirational) (A) | |
|  | # of Australian staff ( D) |  | # of team meetings (D) |  |  | # of peer-reviewed research articles ( R) |  | # of users for whom the new data is available (can be differentiated by sector or organisation) ( R) | |
|  | # of Fijian students involved ( D) |  | obtaining Ethics approval from Fijian authorities (F) |  |  | # of other publications (publicly available) ( R) |  | # of users who use the new data (can be differentiated by sector or organisation) ( A) | |
|  | # of Australian students involved ( D) | Data collection | # of policy documents retrieved ( D) |  |  | # of presentations in international/regional conferences ( R) |  | # of times new data was used as evidence in writing (A ) | |
|  | # of hours allocated to component / staff ( D) |  | # of interviews conducted (D) |  |  | # of presentations in national conferences ( R) | Reach of publications | # of citations (A ) | |
| Collaboration | # of project teams involved ( R) |  | # of interviews conducted with health sector government officials (D) |  |  | # of workshops for international/regional audience ( R) |  | # of downloads (R ) | |
| Capacity building | # training sessions on any component of PLA ( D) |  | # of interviews conducted with non-health sector government officials ( R) |  |  | # of workshops for national audience ( R) |  | # of reads (R ) | |
|  | # of staff trained on any components of PLA (D/R) |  | # of interviews conducted with development partners and NGOs ( R) |  |  | # of attended audience ( R) |  | # of Altmetric score (A ) | |
|  |  |  | # of interviews conducted with other type of actors (e.g. industry) ( R) | Strengthened local (Fijian) research capacity | | # of Fijian staff trained on any aspects of this project stream (D/R) | Reach of outputs in social media | # of media mentions (R ) | |
|  |  |  | # of interview participants (D) |  |  | # of authors from Fiji included on academic publications ( R/D) |  | # of mentions on Twitter (R ) | |
|  |  |  | # of interview participants - health sector government officials (D) |  |  | # of academic publications co-authored by Fijian project staff ( R/D) |  | # of mentions on Facebook (R ) | |
|  |  |  | # of interview participants non-health sector government officials ( R) |  |  | # of PhD degrees earned by Fijian staff members ( R) |  | # of Twitter likes or comments (R ) | |
|  |  |  | # of interview participants - development partners and NGOs ( R) |  |  | # of Masters degrees earned by Fijian staff members ( R) |  | # of Facebook likes or comments (R ) | |
|  |  |  | # of interview participants - other type of actors (e.g. industry) ( R) | Strengthening local (Fijian) economy | | # of jobs created in Fiji ( R) |  | # of website views (R ) | |
|  |  |  | # of staff engaged in collecting documents for PLA (D) |  |  | # of jobs maintained in Fiji ( R) |  |  | |
|  |  |  | # of staff engaged in conducting interviews (D) | Strengthening Australian research capacity | | # of Australian staff trained on any aspects of the PLA stream (D/R) |  |  | |
|  |  | Analysis | # of staff working on analysis ( D) |  |  | # of PhD degrees earned by Fijian staff members ( R) |  |  | |
|  |  |  |  |  |  | # of Masters degrees earned by Fijian staff members ( R) |  |  | |
|  |  |  |  | Strengthening the Australian economy | | # of jobs created in Australia |  |  | |
|  |  |  |  |  |  | # of jobs maintained in Australia |  |  | |
|  |  |  |  | Additional lifetime income | | Amount of additional lifetime income of PhD students |  |  | |

**Table S2B The economic modelling project stream data collection card (Cs – Cost; D – Dose; R – Reach; F – Fidelity; A - Adoption)**

| **Input indicators** | | **Process indicators** | | **Output indicators** | | **Outcome indicators** | |
| --- | --- | --- | --- | --- | --- | --- | --- |
| **Project Stream Component** | **Input indicator** | **Project Stream Component** | **Process indicator** | **Project Stream Component** | **Output indicators** | **Project Stream Component** | **Outcome indicators** |
| Funding | $ spent budget (Cs) | Planning / Coordination | # of internal reports and briefs (D) | New data | Economic modelling data of SSB taxes (F) | Contributing evidence to policy development, scale-up or strengthening | # of times evidence provided in writing (D) |
|  | $ spent on wages of Fijian research staff (Cs) |  | # of team meetings (D) |  | Economic modelling data of salt reduction strategies (F) |  | # policy forums held (D) |
|  | $ spent on wages of Australian research staff (Cs) | Data collection | # of databases included ( R) | Dissemination | Report and summary brief on economic modelling of SSB taxes (F) |  | # of policy makers involved (R ) |
| Human resource | # of Fijian staff ( D) |  | # of staff engaged in data collection? (D) |  | Report and summary brief on economic modelling of salt reduction strategies (F) |  | # changed policies (aspirational) (A) |
|  | # of Australian staff ( D) | Analysis | # days spent with analysis (D) |  | # of participants/stakeholders receiving the summary brief on sugar sweetened beverage tax modelling ( R) |  | # of users for whom the new data is available (can be differentiated by sector or organisation) (R ) |
|  | # of Fijian students involved ( D) |  | # of staff engaged in data analysis (D) |  | # of participants/stakeholders receiving the summary brief on salt reduction strategies modelling taxes ( R) |  | # of users who use the new data (can be differentiated by sector or organisation) (A ) |
|  | # of Australian students involved ( D) | Reporting | # of internal reports and briefs (D) |  | # of peer-reviewed research articles ( R) |  | # of times new data was used as evidence in writing ( A) |
|  | # of hours allocated to component / staff ( D) |  | # of stakeholders receiving interal briefs ( R) |  | # of other publications (publicly available) ( R) | Reach of publications | # of citations ( A) |
| Collaboration | # of project teams involved ( R) | Stakeholder engagement | # stakeholders engaged ( R) |  | # of presentations in international/regional conferences ( R) |  | # of downloads (R ) |
| Capacity building | # staff trained on modelling (D/R) |  | # meetings coordinated to gain information for tax scenarios and what kind of outputs they're interested in ( D) |  | # of presentations in national conferences (R ) |  | # of reads (R ) |
|  |  |  |  |  | # of workshops for international/regional audience (R ) |  | # of Altmetric score ( A) |
|  |  |  |  |  | # of workshops for national audience (R ) | Reach of outputs in social media | # of media mentions (R ) |
|  |  |  |  |  | # of attended audience (R ) |  | # of mentions on Twitter (R ) |
|  |  |  |  | Strengthened local (Fijian) research capacity | # of Fijian staff trained on any aspects of this project stream (D/R) |  | # of mentions on Facebook (R ) |
|  |  |  |  |  | # of authors from Fiji included on academic publications ( R/D) |  | # of Twitter likes or comments (R ) |
|  |  |  |  |  | # of academic publications co-authored by Fijian project staff ( R/D) |  | # of Facebook likes or comments (R ) |
|  |  |  |  |  | # of PhD degrees earned by Fijian staff members ( R) |  | # of website views (R ) |
|  |  |  |  |  | # of Masters degrees earned by Fijian staff members ( R) |  |  |
|  |  |  |  | Strengthening local (Fijian) economy | # of jobs created in Fiji (R ) |  |  |
|  |  |  |  |  | # of jobs maintained in Fiji (R ) |  |  |
|  |  |  |  | Strengthening Australian research capacity | # of Australian staff trained on any aspects of the PLA stream (D/R) |  |  |
|  |  |  |  |  | # of PhD degrees earned by Fijian staff members ( R) |  |  |
|  |  |  |  |  | # of Masters degrees earned by Fijian staff members ( R) |  |  |
|  |  |  |  | Strengthening the Australian economy | # of jobs created in Australia (R ) |  |  |
|  |  |  |  |  | # of jobs maintained in Australia (R ) |  |  |
|  |  |  |  | Additional lifetime income | Amount of additional lifetime income of PhD students (R ) |  |  |

**Table S2C The cross-sectional nutrition survey sub-stream data collection card (Cs – Cost; D – Dose; R – Reach; F – Fidelity; A - Adoption)**

| **Input indicators** | | **Process indicators** | | **Output indicators** | | **Outcome indicators** | | |
| --- | --- | --- | --- | --- | --- | --- | --- | --- |
| **Project Stream Component** | **Input indicator** | **Project Stream Component** | **Process indicator** | **Project Stream Component** | **Output indicators** | **Project Stream Component** | **Outcome indicators** |  |
| Funding | $ spent budget (Cs) | Preparation / Planning / Coordination | # of internal reports and briefs (D) | New data | # of data sets on disaggregated mean levels of sodium and sugar intake levels for adults (F) | Proposed new targets based on new datasets | # of new targets proposed (A) |  |
|  | $ spent on wages of Fijian research staff (Cs) |  | # of stakeholders receiving internal briefs ( R) |  | # of data sets on Knowledge, attitude, behaviour (F) | Adoption of new targets based on new datasets in Fiji | Inclusion of sodium and sugar target levels with deadlines in Ministry of Health and Medical Services plans (A ) |  |
|  | $ spent on wages of Australian research staff (Cs) |  | # of team meetings (D) |  | # of data sets on mean sources of sodium and sugar in diet (F) | Adoption of a new system for monitoring food policy impact in Fiji | New components adopted in the monitoring activity included in Ministry of Health and Medical Services plans ( A) |  |
| Human resources | # of Fijian staff ( D) |  | # of survey data collection applications developed (F) | Dissemination | # of summary briefs on nutrition survey results (F) | Contributing evidence to policy development, scale-up or strengthening | # of users for whom the new data is available (can be differentiated by sector or organisation) ( R) |  |
|  | # of Australian staff ( D) |  | obtaining Ethics approval from Fijian authorities and UNSW Human Ethics Committee (F) |  | # of participants/stakeholders receiving the summary brief ( R) |  | # of users who use the new data (can be differentiated by sector or organisation) ( A) |  |
|  | # of Fijian students involved ( D) | Data collection | # of participants screened ( R) |  | # of peer-reviewed research articles ( R) |  | # of times new data was used as evidence in writing (A) |  |
|  | # of Australian students involved ( D) |  | # of staff participating in data collection (D) |  | # of other publications (publicly available) ( R) | Reach of publications | # of citations (A ) |  |
|  | # of hours allocated to component / staff ( D) |  | # of hours/days spent with data collection (D) |  | # of FNU newsletters and media releases ( R) |  | # of downloads ( R) |  |
| Collaboration | # of project teams involved ( R) |  | # FJD/AUD provided to survey participants (Cs) |  | # of individuals receiving the newsletter ( R) |  | # of reads ( R) |  |
|  | # of collaborations external to the project teams ( R) |  | # FJD/AUD spent on field expenses of data collection (Cs) |  | # of media releases in local newspapers ( R) |  | # of Altmetric score (A) |  |
| Capacity building | # of Fijian research assistants trained on any aspect of this project stream ( R/D) | Community engagement | # of meetings with communities (D) |  | # of media releases in non-local newspapers or media platforms ( R) | Reach of outputs in social media | # of media mentions ( R) |  |
|  | # of training sessions delivered on any aspect of this project stream (D) |  | # of communities met with ( R) |  | # of presentations in international/regional conferences ( R) |  | # of mentions on Twitter ( R) |  |
| Assets | # of tablets (Samsung tablets) (D) |  | # of phone calls and emails received over the support lines ( R) |  | # of presentations in national conferences ( R) |  | # of mentions on Facebook ( R) |  |
|  | # of software license (D) |  |  |  | # of workshops for international/regional audience ( R) |  | # of Twitter likes or comments ( R) |  |
|  | # of weighing scales (D) |  |  |  | # of workshops for national audience ( R) |  | # of Facebook likes or comments ( R) |  |
|  | # of blood pressure monitors (D) |  |  |  | # of attended audience ( R) |  | # of website views ( R) |  |
|  | # of stadiometers (D) |  |  | Strengthened public health system | New guideline developed for strengthening monitoring activity, input for collaborative process to strengthen policy development and implementation (F) |  |  |  |
|  |  |  |  | Strengthened local (Fijian) research capacity | # of Fijian staff trained on any aspects of this project stream (D/R) |  |  |  |
|  |  |  |  |  | # of authors from Fiji included on academic publications ( R/D) |  |  |  |
|  |  |  |  |  | # of academic publications co-authored by Fijian project staff ( R/D) |  |  |  |
|  |  |  |  |  | # of PhD degrees earned by Fijian staff members ( R) |  |  |  |
|  |  |  |  |  | # of Masters degrees earned by Fijian staff members ( R) |  |  |  |
|  |  |  |  | Strengthening local (Fijian) economy | # of jobs created in Fiji ( R) |  |  |  |
|  |  |  |  |  | # of jobs maintained in Fiji ( R) |  |  |  |
|  |  |  |  | Strengthening Australian research capacity | # of Australian staff trained on any aspects of the PLA stream (D/R) |  |  |  |
|  |  |  |  |  | # of PhD degrees earned by Fijian staff members ( R) |  |  |  |
|  |  |  |  |  | # of Masters degrees earned by Fijian staff members ( R) |  |  |  |
|  |  |  |  | Strengthening the Australian economy | # of jobs created in Australia ( R) |  |  |  |
|  |  |  |  |  | # of jobs maintained in Australia ( R) |  |  |  |
|  |  |  |  | Additional lifetime income | Amount of additional lifetime income of PhD students ( R) |  |  |  |
