# Supplementary material for: Research protocol for impact assessment of a project to scale up food policies in the Pacific
Source: Health Res Policy Syst. 2022 Oct 29;20:117. doi: 10.1186/s12961-022-00927-x (PMC9617745; doi:10.1186/s12961-022-00927-x)
Supplement: Supplementary file 3 — Additional file 3. Interview guide for the research impact assessment. [file 12961_2022_927_MOESM3_ESM.docx]

**Additional File 3. Interview Guide**

**Research Impact Assessment of the Scaling-up food policy interventions to reduce noncommunicable diseases in the Pacific Islands**

**PARTICIPANT INFORMATION**

| **Country** |  |
| --- | --- |
| **Participant Identifier Number** |  |
| **Date** |  |

**BACKGROUND**

**Participants will be asked about** **the implementation and the impact of the ‘Scaling-up food policy interventions to reduce noncommunicable diseases in the Pacific Islands’ research project (SUPI). In addition, they will be asked to answer questions relating to specific project components or interventions that they have been involved in.**

**INTRODUCTION**

**Thank you for taking the time to join our interview. The aim of this group is to discuss the activities and impact of the scaling-up food policy interventions project in Fiji. We want to get your opinions about the different components of this project and how useful they have been.**

**We would also like to remind you that this discussion is being audio-recorded. Any personally identifiable information is removed during transcription, and responses are made anonymous.**

**INTERVIEW TOPICS**

**Part 1. Research impact**

**Theme 1: Strengthened public health system and policy**

Example questions:

1. Is SUPI on track to achieve its aim to strengthen the public health system and policy?
2. Are we on track to create the necessary outputs to achieve this aim? What are the barriers and enablers?
3. What are the mechanisms through what we achieve change in this domain?

**Theme 2: Community and health benefits & beneficial economic impact**

Example questions:

1. How do our current activities help reducing the salt and sugar content of commonly consumed foods and improve dietary patterns in Fiji? How do these changes impact the Fijian economy?
2. What are the barriers and enablers to achieve this impact?
3. What are the mechanisms through what we achieve change in this domain?

**Theme 3: Knowledge advancement**

Example questions:

1. In what ways has SUPI strengthened knowledge on planning and implementing effective food policies in Fiji/in other PICs/in LMICs?
2. Are we achieving the extent of knowledge advancement as planned? What are the barriers and enablers?
3. What are the mechanisms through what we achieve change in this domain?

**Theme 4: Strengthened research capacity and capability**

Example questions:

1. In what ways has SUPI strengthened Fijian and Australian research capacity and capability on implementation science projects in food policy?
2. Are we achieving the extent of capacity and capability as planned? What are the barriers and enablers?
3. What are the mechanisms through what we achieve change in this domain?

**Part 2. Process evaluation**

**Participants will be questioned on topics relevant to their roles within SUPI, specific for the following project streams:**

**Policy landscape analysis**

| Dimension | Interview questions |
| --- | --- |
| Fidelity | Were all project components delivered as planned? If not, why not? (Were the aims/actions/roles clear? Were adequate staff/skill/time/assets allocated?) |
| Effectiveness | Do you think that this project stream was implemented effectively? What could have been done to make the implementation more effective? |
|  | Have the outputs of the PLA achieved the intended aim to support the policy strengthening component of the project? |
| Context | Were there any components within this project stream that were particularly challenging/easy to deliver? If yes, why? What were the facilitators/barriers affecting the implementation of this project stream? |
| Mechanisms of change | In what ways can/have the outputs of this project stream induce(d) policy change? |

**Economic modelling project stream**

| Dimension | Interview questions |
| --- | --- |
| Fidelity | Were all project components delivered as planned? If not, why not? (Were the aims/actions/roles clear? Were adequate staff/skill/time/assets allocated?) |
| Effectiveness | Do you think that this project stream was implemented effectively? What could have been done to make the implementation more effective? |
|  | Have the outputs of the economic modelling achieved the intended aim to support the policy strengthening component of the project? |
| Context | Were there any components within this project stream that were particularly challenging/easy to deliver? If yes, why? What were the facilitators/barriers affecting the implementation of this project stream? |
| Mechanisms of change | In what ways can/have the outputs of this project stream induce(d) policy change? |

**Nutrition survey project stream**

| Dimension | Interview questions |
| --- | --- |
| Fidelity | Were all project components delivered as planned? If not, why not? (Were the aims/actions/roles clear? Were adequate staff/skill/time/assets allocated?) |
| Effectiveness | Do you think that this project stream was implemented effectively? What could have been done to make the implementation more effective? |
|  | Have the outputs of the nutrition survey achieved the intended aim to support the policy strengthening component of the project and monitor the impact of food and nutrition interventions? |
| Context | Were there any components within this project stream that were particularly challenging/easy to deliver? If yes, why? What were the facilitators/barriers affecting the implementation of this project stream? |
| Mechanisms of change | In what ways can/have the outputs of this project stream induce(d) policy change? |

**Food composition survey stream**

| Dimension | Interview questions |
| --- | --- |
| Fidelity | Were all project components delivered as planned? If not, why not? (Were the aims/actions/roles clear? Were adequate staff/skill/time/assets allocated?) |
| Effectiveness | Do you think that this project stream was implemented effectively? What could have been done to make the implementation more effective? |
|  | Have the outputs of the food composition assessment achieved the intended aim to support the policy strengthening component of the project and the monitoring of food and nutrition interventions? |
| Context | Were there any components within this project stream that were particularly challenging/easy to deliver? If yes, why? What were the facilitators/barriers affecting the implementation of this project stream? |
| Mechanisms of change | In what ways can/have the outputs of this project stream induce(d) policy change? |

**Policy development and implementation strengthening project stream**

| Dimension | Interview questions |
| --- | --- |
| Fidelity | Were all project components delivered as planned? If not, why not? |
| Dose/ Adoption | How well do stakeholders engage with the activities of this project stream? |
| Effectiveness | Do you think that this project stream was implemented effectively? What could have been done to make the implementation more effective? |
|  | Have the outputs of the collaborative process to strengthen policy development and implementation achieved the intended aim to support the policy strengthening component of the project? |
| Context | Were there any components within this project stream that were particularly challenging to deliver? If yes, why? |
| Mechanisms of change | In what ways can/have the outputs of this project stream induce(d) policy change? |
