# Supplementary material for: Research protocol for impact assessment of a project to scale up food policies in the Pacific
Source: Health Res Policy Syst. 2022 Oct 29;20:117. doi: 10.1186/s12961-022-00927-x (PMC9617745; doi:10.1186/s12961-022-00927-x)
Supplement: Supplementary file 4 — Additional file 4. Topic guide for the group discussions for the research impact assessment. [file 12961_2022_927_MOESM4_ESM.docx]

**Additional File 4. Topic Guide for Group Discussions**

**Research Impact Assessment of the Scaling-up food policy interventions to reduce noncommunicable diseases in the Pacific Islands**

**DISCUSSION GROUP INFORMATION**

| **Country** |  |
| --- | --- |
| **Discussion Group Number** |  |
| **Stakeholder Organisations/Sectors represented in focus group (i.e., industry, health, etc.)** |  |
| **Date** |  |

**BACKGROUND**

**Participants will be asked to the implementation and the impact of the ‘Scaling-up food policy interventions to reduce noncommunicable diseases in the Pacific Islands’ project (SUPI). In addition, they will be asked to answer questions relating to specific project components or interventions that they have been involved in.**

**INTRODUCTION**

**Thank you for taking the time to join our group discussion. The aim of this group is to discuss the activities and impact of the scaling-up food policy interventions project in Fiji. We want to get your opinions about the different components of this project and how useful they have been.**

**We would also like to remind you that this discussion is being audio-recorded.**

**DISCUSSION GROUP TOPICS**

**Theme 1: Strengthened public health system and policy**

Example questions:

1. Is SUPI on track to achieve its aim to strengthen the public health system and policy? What programs and policies have been strengthened and in what ways?
2. What are the mechanisms through what we achieve change in this domain?
3. Are we on track to create the necessary outputs to achieve this aim? What are the barriers and enablers?
4. Are we engaging with key stakeholders adequately? If not, what could we do better?

**Theme 2: Community and health benefits & beneficial economic impact**

Example questions:

1. How do our current activities help reducing the salt and sugar content of commonly consumed foods and improve dietary patterns in Fiji? How do these changes impact the Fijian economy?
2. What are the mechanisms through what we achieve change in this domain?
3. What are the barriers and enablers to achieve this impact?
4. Are there any other programs/initiatives currently in Fiji that would enhance or constrain our efforts to achieve this impact?
5. What else could we do to achieve this impact?

**Theme 3: Knowledge advancement**

Example questions:

1. In what ways has SUPI strengthened knowledge on planning and implementing effective food policies in Fiji/in other PICs/in LMICs?
2. What are the mechanisms through what we achieve change in this domain?
3. Are we achieving the extent of knowledge advancement as planned? What are the barriers and enablers?
4. What could we do to strengthen this impact?

**Theme 4: Strengthened research capacity and capability**

Example questions:

1. In what ways has SUPI strengthened Fijian and Australian research capacity and capability on implementation science projects in food policy?
2. What are the mechanisms through what we achieve change in this domain?
3. Are we achieving the extent of capacity and capability as planned? What are the barriers and enablers?
4. What could we do to further strengthen this impact?
