# Supplementary material for: Research protocol for impact assessment of a project to scale up food policies in the Pacific
Source: Health Res Policy Syst. 2022 Oct 29;20:117. doi: 10.1186/s12961-022-00927-x (PMC9617745; doi:10.1186/s12961-022-00927-x)
Supplement: Supplementary file 5 — Additional file 5. Research impact assessment quantitative summary score card. [file 12961_2022_927_MOESM5_ESM.docx]

**Additional File 5. Research impact assessment: Quantitative Summary Score Card**

*The impact values are automatically accommodated as the project stream data collection cards are filled out. N/A indicates whether the given project stream feeds into that domain of benefit metrics. At this stage, we do not know yet which interventions will be chosen by the Fijian government that we will support, thus the process evaluation indicators will need to be identified at a later stage.

Table S5A Research impact assessment, summary table of quantitative analysis, part 1

|  | **Metrics** | | **Impact Value*** | | | | | | | | | | |
| --- | --- | --- | --- | --- | --- | --- | --- | --- | --- | --- | --- | --- | --- |
| **Domains of Benefit** | **Metric** | **Indicator** | Stream 1 | Stream 2 | Stream 4 (Nutrition survey) | Stream 4 (Food composition survey) | Stream 3 | Intervention 1 | Intervention 2 | Intervention 3 | General (not elsewhere stated) | Total Impact |  |
| **Public Health System & Policy Strengthening** | Adoption of a new system for monitoring food policy impact in Fiji | New components adopted in the monitoring activity included in Ministry of Health and Medical Services plans | N/A | N/A | 0 | N/A | 0 |  |  |  | N/A | 0 |  |
|  | Adoption of new targets based on new datasets in Fiji | Inclusion of sodium and sugar intake target levels with deadlines in Ministry of Health and Medical Services plans | N/A | N/A | 0 | N/A | 0 |  |  |  | N/A | 0 |  |
|  | Changes to current food policies in Fiji | # of changed policies or plans (aspirational) | 0 | 0 | 0 |  | 0 |  |  |  | N/A | 0 |  |
|  | New school policies on food implemented in Fijian schools | # of schools implementing new (or existing) food policies to target the consumption and availability of unhealthy foods in schools (based on intervention) |  |  |  |  |  |  |  |  |  | 0 |  |
| **Community and Health Benefits** | Decrease in NCD prevalence and associated risk factors in Fiji | Decrease in the prevalence of hypertension and cardiovascular diseases among adults (%) | N/A | N/A | N/A | N/A | N/A | *Based on interventions* | | | N/A | 0 |  |
|  |  | Change in average age of disease onset for hypertension and cardiovascular diseases (%) | N/A | N/A | N/A | N/A | N/A | *Based on interventions* | | | N/A | 0 |  |
|  |  | Decrease in the prevalence of diabetes among adults (%; +/- increase in age of disease onset) | N/A | N/A | N/A | N/A | N/A | *Based on interventions* | | | N/A | 0 |  |
|  |  | Change in average age of disease onset for diabetes (%) | N/A | N/A | N/A | N/A | N/A | *Based on interventions* | | | N/A | 0 |  |
|  |  | Decrease in the prevalence of obesity and overweight among adults (%) | N/A | N/A | N/A | N/A | N/A | *Based on interventions* | | | N/A | 0 |  |
|  | Consumer knowledge and awareness of health risks associated with salt and sugar consumption | % of improvement in Knowledge (as part of KAB data) | N/A | N/A | N/A | N/A | N/A | *Based on interventions* | | | N/A | 0 |  |
|  |  | % of improvement in Attitudes (as part of KAB data) | N/A | N/A | N/A | N/A | N/A | *Based on interventions* | | | N/A | 0 |  |
|  |  | % of improvement in Behaviour (as part of KAB data) | N/A | N/A | N/A | N/A | N/A | *Based on interventions* | | | N/A | 0 |  |
|  | Reduced affordability of processed foods | % of absolute/relative change in processed food prices | N/A | N/A | N/A | N/A | N/A | *Based on interventions* | | | N/A | 0 |  |
|  | Reduced availability of processed foods | # of high sodium/high sugar products readily available in schools, supermarkets, workplaces | N/A | N/A | N/A | N/A | N/A | *Based on interventions* | | | N/A | 0 |  |
|  | Reduced consumption of processed foods | % of reduction in processed foods consumption | N/A | N/A | N/A | N/A | N/A | *Based on interventions* | | | N/A | 0 |  |
|  | Product reformulation | % of decrease in sodium content of processed food products | N/A | N/A | N/A | N/A | N/A | *Based on interventions* | | | N/A | 0 |  |
|  |  | % of decrease in sugar content of processed food products | N/A | N/A | N/A | N/A | N/A | *Based on interventions* | | | N/A | 0 |  |
|  | Reduction in salt/sugar intake | % of reduction in daily sodium intake | N/A | N/A | N/A | N/A | N/A | *Based on interventions* | | | N/A | 0 |  |
|  |  | % of reduction in daily sugar intake | N/A | N/A | N/A | N/A | N/A | *Based on interventions* | | | N/A | 0 |  |

Table S5B Research impact assessment, summary table of quantitative analysis, part 2

|  | **Metrics** | | **Impact Value*** | | | | | | | | | |
| --- | --- | --- | --- | --- | --- | --- | --- | --- | --- | --- | --- | --- |
| **Domains of Benefit** | **Metric** | **Indicator** | Stream 1 | Stream 2 | Stream 4 (Nutrition survey) | Stream 4 (Food composition survey) | Stream 3 | Intervention 1 | Intervention 2 | Intervention 3 | General (not elsewhere stated) | Total Impact |
| **Economic Impact** | Current and future income of staff associated with the study | Amount of research team wages contributed to the Fijian economy: (total wages) | $ - | $ - | $ - | $ - | $ - |  |  |  |  | 0 |
|  |  | # of Fijian research staff receiving wage for participation in this project | 0 | 0 | 0 | 0 | 0 |  |  |  |  |  |
|  |  | Amount of research team wages contributed to the Australian economy: (total wages) | $ - | $ - | $ - | $ - | $ - |  |  |  |  | 0 |
|  |  | # of Australian research staff receiving wage for participation in this project | 0 | 0 | 0 | 0 | 0 |  |  |  |  | 0 |
|  |  | # of jobs created in Fiji | 0 | 0 | 0 | 0 | 0 |  |  |  |  | 0 |
|  |  | # of jobs maintained in Fiji | 0 | 0 | 0 | 0 | 0 |  |  |  |  | 0 |
|  |  | # of jobs created in Australia | 0 | 0 | 0 | 0 | 0 |  |  |  |  | 0 |
|  |  | # of jobs maintained in Australia | 0 | 0 | 0 | 0 | 0 |  |  |  |  | 0 |
|  |  | Amount of additional lifetime income of PhD students | $ - | $ - | $ - | $ - | $ - |  |  |  |  | 0 |
|  | Reduced health system costs and generated revenues (depends on selected initiatives) | Amount of revenue generated with x% raise of SSB tax (economic modelling of sugar sweetened beverage taxes in Fiji) (could be economic or policy/legislation) | N/A | N/A | N/A | N/A | N/A | Based on interventions | | | N/A | 0 |
|  |  | Amount saved with implementing x interventions (economic modelling of salt reduction strategies in Fiji) | N/A | N/A | N/A | N/A | N/A | Based on interventions | | | N/A | 0 |
|  | Reduced spending on processed foods | Estimate can be calculated based on price data and % of consumption change, estimated for the population based on the cohort data | N/A | N/A | N/A | N/A | N/A | Based on interventions | | | N/A | 0 |
|  | New research financing | Amount of new research funds gained (leveraged funding) | N/A | N/A | N/A | N/A | N/A | N/A | N/A | N/A | $ | 0 |
| **Knowledge Advancement** | New data (sets) | # of new data sets | 1 | 2 | 0 | 0 | N/A | N/A | N/A | N/A | N/A | 3 |
|  |  | # of users for whom the new data is available (can be differentiated by sector or organisation) | 0 | 0 | 0 | 0 | N/A | N/A | N/A | N/A | N/A | 0 |
|  |  | # of users who use the new data (can be differentiated by sector or organisation) | 0 | 0 | 0 | 0 | N/A | N/A | N/A | N/A | N/A | 0 |
|  |  | # of times new data was used as evidence in writing | 0 | 0 | 0 | 0 | N/A | N/A | N/A | N/A | N/A | 0 |
|  | Publications (publicly available) | # of peer-reviewed research articles | 0 | 0 | 0 | 0 | 0 |  |  |  | N/A | 0 |
|  |  | # of other publications (publicly available) | 0 | 0 | 0 | 0 | 0 |  |  |  | N/A | 0 |
|  |  | # of citations | 0 | 0 | 0 | 0 | 0 |  |  |  | N/A | 0 |
|  |  | # of downloads | 0 | 0 | 0 | 0 | 0 |  |  |  | N/A | 0 |
|  |  | # of reads | 0 | 0 | 0 | 0 | 0 |  |  |  | N/A | 0 |
|  |  | # of Altmetric score | 0 | 0 | 0 | 0 | 0 |  |  |  | N/A | 0 |
|  | Newsletters | # of newsletters | N/A | N/A | 0 | N/A | N/A |  |  |  | N/A | 0 |
|  |  | # of individuals received the newsletters | N/A | N/A | 0 | N/A | N/A |  |  |  | N/A | 0 |
|  | Results briefs & technical documents | # of results brief | 1 | 2 | 0 | 0 | 1 |  |  |  | N/A | 4 |
|  |  | # of individuals received the briefs | 0 | 0 | 0 | 0 | 0 |  |  |  | N/A | 0 |
|  | Presentations, webinars, workshops | # of presentations in international/regional conferences | 0 | 0 | 0 | 0 | 0 |  |  |  | N/A | 0 |
|  |  | # of presentations in national conferences | 0 | 0 | 0 | 0 | 0 |  |  |  | N/A | 0 |
|  |  | # of workshops for international/regional audience | 0 | 0 | 0 | 0 | 0 |  |  |  | N/A | 0 |
|  |  | # of workshops for national audience | 0 | 0 | 0 | 0 | 0 |  |  |  | N/A | 0 |
|  |  | # of attended audience | 0 | 0 | 0 | 0 | 0 |  |  |  | N/A | 0 |
|  | Media and Social Media | # of media mentions | 0 | 0 | 0 | 0 | 0 |  |  |  | N/A | 0 |
|  |  | # of mentions on Twitter | 0 | 0 | 0 | 0 | 0 |  |  |  | N/A | 0 |
|  |  | # of mentions on Facebook | 0 | 0 | 0 | 0 | 0 |  |  |  | N/A | 0 |
|  |  | # of Twitter likes or comments | 0 | 0 | 0 | 0 | 0 |  |  |  | N/A | 0 |
|  |  | # of Facebook likes or comments | 0 | 0 | 0 | 0 | 0 |  |  |  | N/A | 0 |
|  |  | # of website views | 0 | 0 | 0 | 0 | 0 |  |  |  | N/A | 0 |

Table S5C Research impact assessment, summary table of quantitative analysis, part 3

|  | **Metrics** | | **Impact Value*** | | | | | | | | | |
| --- | --- | --- | --- | --- | --- | --- | --- | --- | --- | --- | --- | --- |
| **Domains of Benefit** | **Metric** | **Indicator** | Stream 1 | Stream 2 | Stream 4 (Nutrition survey) | Stream 4 (Food composition survey) | Stream 3 | Intervention 1 | Intervention 2 | Intervention 3 | General (not elsewhere stated) | Total Impact |
| **Research Capacity and Capability Building** | Academic qualifications | # of Fijian students associated with the project | 0 | 0 | 0 | 0 | 0 |  |  |  | N/A | 0 |
|  |  | # of Australian students associated with the project | 0 | 0 | 0 | 0 | 0 |  |  |  | N/A | 0 |
|  |  | # of Masters degrees earned by Fijian staff members | 0 | 0 | 0 | 0 | N/A |  |  |  | N/A | 0 |
|  |  | # of PhD degrees earned by Fijian staff members | 0 | 0 | 0 | 0 | N/A |  |  |  | N/A | 0 |
|  | Researchers and Research Assistants | # of researchers and research assistants with capacity built in implementation science (any aspect of the project) | 0 | 0 | 0 | 0 | 0 |  |  |  | N/A | 0 |
|  | Knowledge and capabilities in developing and implementing food and nutrition policies | # of Fijian researchers and government officials participated in any aspect of the project | 0 | 0 | 0 | 0 | 0 |  |  |  | N/A | 0 |
|  | New research network | # of Fijian staff who are co-authors on peer-reviewed papers | 0 | 0 | 0 | 0 | 0 |  |  |  | N/A | 0 |
|  |  | # of Fijian staff who are collaborators on future grants | N/A | N/A | N/A | N/A | N/A | N/A | N/A | N/A | 0 | 0 |
|  |  | # of co-applications for future grants | N/A | N/A | N/A | N/A | N/A | N/A | N/A | N/A | 0 | 0 |
